# Fabrication of three-dimensional hydrogel scaffolds for modeling shunt failure by tissue obstruction in hydrocephalus

**DOI:** 10.1186/s12987-015-0023-9

**Published:** 2015-11-14

**Authors:** Carolyn Harris, Kelsie Pearson, Kristen Hadley, Shanshan Zhu, Samuel Browd, Brian W. Hanak, William Shain

**Affiliations:** Center for Integrative Brain Research, Seattle Children’s Research Institute, Seattle, WA 98101 USA; Department of Neurosurgery, Wayne State University, 3901 Beaubien Blvd, 2nd Floor Carls Building, Detroit, MI 48201 USA; Department of Neurological Surgery, University of Washington, 1959 NE Pacific Street, Seattle, WA 98195 USA

**Keywords:** Hydrocephalus, Shunt obstruction, In vitro modeling, Three-dimensional scaffold, Alginate

## Abstract

**Background:**

Shunt obstruction in the treatment of hydrocephalus is poorly understood, is multi-factorial, and in many cases is modeled ineffectively. Several mechanisms may be responsible, one of which involves shunt infiltration by reactive cells from the brain parenchyma. This has not been modeled in culture and cannot be consistently examined in vivo without a large sample size.

**Methods:**

We have developed and tested a three-dimensional in vitro model of astrocyte migration and proliferation around clinical grade ventricular catheters and into catheter holes that mimics the development of cellular outgrowth from the parenchyma that may contribute to shunt obstruction.

**Results:**

Cell attachment and growth was observed on shunt catheters for as long as 80 days with at least 77 % viability until 51 days. The model can be used to study cellular attachment to ventricular catheters under both static and pulsatile flow conditions, which better mimic physiological cerebrospinal fluid dynamics and shunt system flow rates (0.25 mL/min, 100 pulses/min). Pulsatile flow through the ventricular catheter decreased cell attachment/growth by 63 % after 18 h. Under both conditions it was possible to observe cells accumulating around and in shunt catheter holes.

**Conclusions:**

Alone or in combination with previously-published culture models of shunt obstruction, this model serves as a relevant test bed to analyze mechanisms of shunt failure and to test catheter modifications that will prevent cell attachment and growth.

## Background

Hydrocephalus, an imbalance between cerebrospinal fluid (CSF) production and absorption, is a chronic disorder causing abnormal enlargement of the cerebral ventricles and ventriculomegaly. Since 1955, the standard treatment for hydrocephalus has been the surgical placement of shunts to drain CSF [1]. However, pediatric shunts have one of the highest failure rates among neurological devices; 40 % of shunts fail within 2 years of implantation [2]. Hospitalizations for hydrocephalus have reached 70,000 per year in the USA. Nearly all patients (98 %) who have hydrocephalus will experience shunt failure in their lifetime and most will undergo multiple shunt failures necessitating surgical revision [3]. The societal cost of shunt failure is significant, accounting for the bulk of more than $1 billion annual cost of hydrocephalus care in the USA, not to mention the patient morbidity and mortality associated with shunt dysfunction [4].

Approximately 70 % of shunt failures in the pediatric population in the United States are caused by tissue obstructing the proximal catheter in the ventricles, restricting or preventing CSF outflow [5–9]. In the adult population, obstruction dominates in the valve or distal catheter; the mechanisms behind this mismatch are not well understood. Previous studies have reported a variety of cell types and tissues present on obstructed ventricular catheters comprising connective tissue, choroid plexus, glia-dense tissue masses, ependyma, and pathological cells/tissues including necrotic cellular debris, foreign body giant cells, granulomatous cells, and activated microglia/macrophages and astrocytes [10]. Recent evidence suggests that astrocytes and microglia are the dominant cell types found directly bound to shunt ventricular catheters [11, 12]. Obstructed shunt valves have similar cells bound to them, including glial cells, macrophages/giant cells, and lymphomonocytic cells [11]. While the mechanisms causing ventricular catheter shunt obstruction and down-stream shunt valve obstruction are likely multi-faceted, inflammatory cells certainly play a major role in shunt obstruction in pediatric and adult patients. Perhaps mechanisms including single cell attachment, attachment of migrating and/or proliferating cells suspended in CSF, and/or attachment of activated glia from the brain parenchyma contribute to inflammatory-derived shunt obstruction. [11, 13]. An activated response of astrocytes and microglia is likely to be involved through repair processes stimulated bycatheter insertion and continued catheter presence (i.e. a “foreign-body” response).

Because obstruction of the shunt system is a multimodal problem, high intrinsic variability in clinical and in vivo studies require large numbers of observations, making these time-consuming and cost-prohibitive. In vitro modeling provides opportunities for more precise control of test variables, more rapid throughput, and more consistency of cell growth, but typically lacks the complexity found in the brain (for example, three-dimensional (3D) ventricular and brain components with fluid flow). Novel in vitro modeling can, in this way, provide insight into the mechanisms of inflammatory cell attachment leading to shunt catheter obstruction. In a previous study, an effective in vitro system was developed to understand how single cells suspended in CSF adhere to catheters and contribute to obstruction [14]. In this study, we describe and characterize a 3D in vitro model that mimics shunt failures that occur secondary to cells from the brain parenchyma migrating along the catheter and attaching to the CSF-intake holes, ultimately resulting in obstruction. Our objective was to construct a model for studying shunt catheter hole obstruction by reactive glia from brain parenchyma, one likely mechanism of obstruction. This obstruction could result from either a change in catheter position within the ventricular system, or migration of cells down the catheter into the catheter holes [10]. In this model, cells from a scaffold molded around a clinical ventricular catheter, attach to the catheter in the scaffold, migrate along the external wall and, subsequently attach to and accumulate at shunt catheter holes. By using short- and long- chambers where CSF-intake holes are partially or totally exposed to the CSF, the system models migration of activated astrocytes and microglia out of the brain onto the shunt extending into the ventricle when the catheter is sub-optimally placed, or when placed in its intended location. This 3D hydrocephalus bioreactor provides opportunities to make observations and measurements of cell growth over time with or without flow through the catheter, and provides an efficient test-bed to study modifications to shunt design or surface modifications to improve shunt performance.

## Methods

### Astrocyte cell isolation and maintenance

Procedures were approved by the Institutional Animal Care and Use Committee of Seattle Children’s Research Institute in accordance with the National Institutes of Health Guide for Care and Use of Laboratory Animals. Cortical hemispheres were removed from 3-day old rat pups after deep anesthesia was achieved using inhaled isofluorane. Tissue was cut into 1-mm^3^ pieces and suspended in calcium-free Hanks balanced salt solution (Ca^2+^-HBSS, Invitrogen, Carlsbad, CA, USA). Astrocytes were cultured from these cortical hemispheres using previously published techniques [14]. Briefly, following dissection and mincing, the Ca^2+^-HBSS was removed and replaced by dissociation medium containing 0.25 % trypsin-1X EDTA and incubated for 45 min at 37 °C with 5 % CO_2_ in a 50-mL centrifuge tube. Fresh culture media was then added to quench the trypsin-activity and the tube centrifuged for 3 min at 1300 rpm to collect cells and tissue fragments (primary pellet). The resulting supernatant was collected, transferred into a new centrifuge tube, and centrifuged again to recover any cells that were not collected in the primary pellet. The primary pellet was cycled through a second dissociation and collection process to maximum cell recovery. The primary pellet and the supernatant pellets were then combined, re-suspended, counted, and plated onto T-175 flasks (Invitrogen) at a density of 100,000 cells/mL, or approximately 1,000,000 cells/flask. Cultures were incubated at 37 °C with 5 % CO_2_. Complete culture medium consisted of DMEM (Dulbecco’s Modified Eagle Medium) supplemented with 4.5 g/L d-glucose, 584 mg/L l-glutamine, 110 mg/L sodium pyruvate (Invitrogen), 5 % fetal bovine serum (Invitrogen), 2 % penicillin–streptomycin (Invitrogen), and 0.001 % gentamicin (Sigma, St. Louis, MO, USA). When cells in primary culture reached 80 % confluence, cells not adherent to the culture flask were removed and the adherent astrocytes were dissociated from the flask surface using 0.25 % trypsin-1X EDTA. After collecting the cells by centrifugation and re-suspension in complete medium, cells were passaged re-plated, loaded into scaffolds, or were frozen using Fisher’s CryoMed Controlled-Rate Freezer (Thermo Fisher Scientific, Waltham, MA, USA) and stored in liquid N_2_. All cells used in these studies were replated no more than three times. Astrocyte cultures were fed three times/week.

### Alginate scaffold construction

#### Alginate preparation

Peptide-modified alginate was used to construct a 3D scaffold around shunt ventricular catheters. These scaffolds can support cell attachment and growth [15–18]. Alginate is a natural polysaccharide cross-linked in the presence of Ca^2+^ without the addition of cytotoxic agents [17, 18]. Gel construction followed previously described methods [16, 18, 19]. A 1 % (w/v) sodium alginate (Sigma) solution in 0.3 M NaCl and 0.1 M 2-(*N*-morpholino)ethanesulfonic acid hydrate (MES buffer) was initially functionalized with an RGD (arginine-glycine-aspartic acid) peptide (GGGGRGDY) to improve cell attachment and viability [17, 18, 20]. For standard carbodiimide crosslinking chemistry, the following reagents were added (in order) and incubated for 20 h at room temperature: 8.22 g/L sulfo-*N*-hydroxysulfosuccinimide, 16.44 g/L 1-ethyl-3-[3-dimethylaminopropyl]carbodiimide hydrochloride, 0.3 g/L RGD peptide, and 10 g/L sodium alginate. The reaction was quenched by the addition of 13 g/L hydroxyl amine. The functionalized alginate was then dialyzed using a 3500 molecular weight cutoff snakeskin dialysis membrane (Sigma). The starting dialysis solution contained 7.5 g NaCl/L Milli-Q dIH_2_O. The NaCl content was gradually reduced by 1.25 g/L every 8 h until dilution resulted in removal of all NaCl. After dialysis, a 30-min charcoal filtration treatment was performed. The sample was then lyophilized at 2000 × 10^−3^ mBar at −50 °C for 5 days. Prior to scaffold formation, a 5 % stock of functionalized alginate was prepared in Ca^2+^-HBSS.

#### Chamber preparation

**Fig. 1:**
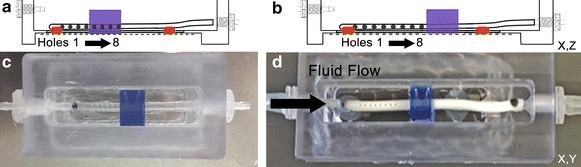
Three-dimensional hydrogel scaffold to model shunt obstruction by brain parenchyma. Representations of short-chambered (**a**, **c**) and long-chambered (**b**, **d**) perfusion systems. **a**, **b** Illustrations of cross-sections through the x,z plane provide details of the relative positions of the alginate scaffolds (*blue profiles*) and catheters. **c**, **d** Photographs of chambers provide views looking down on completed chambers. Short-chambered systems were first used with placement of the alginate scaffold over the four distal ventricular catheter CSF intake holes (**a**, **c**). Long-chambered systems were designed to enable alginate scaffolds to be placed in a more proximal position permitting all of the intake holes to be exposed to the perfusion system “ventricular” space (**b**, **d**). In a subset of experiments, media was pumped into the chamber, flowing to the outside of the catheter and through the catheter holes, then flowing through the inside of the catheter and out of the chamber and catheter (see flow dependent attachment and growth subsection)

**Fig. 2:**
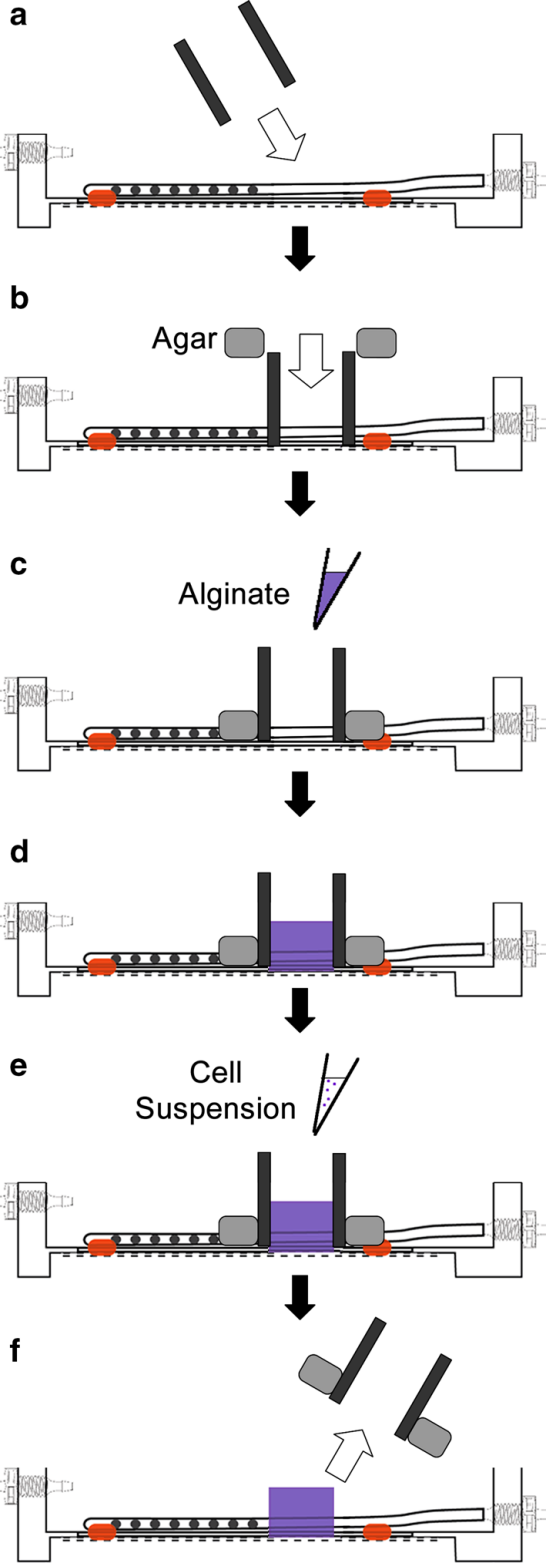
Scaffold construction: schematic of the system modeling the behavior of reactive cellular responses of astrocytes migrating from the parenchyma out to the shunt catheter. Astrocytes were injected into an alginate gel scaffold built around the distal portion of a clinical grade ventricular catheter. Scaffold preparation begins with securing a restraining polystyrene dam around the ventricular portion of the shunt catheter (**a**). The dam is sealed using low-melting point agarose (**b**). Alginate and crosslinkers are injected into the space around the catheter (**c**) and cured for 30 min (**d**). The scaffolds are then injected with a cell suspension resulting in a density of astrocytes similar to that observed in rat cortical tissue (**e**). The dam is removed (**f**) and incubation begins permitting cell migration through the scaffold and onto the catheter

Chambers were assembled using an open polycarbonate base with an inlet and outlet port, a glass coverslip bottom secured with silicone glue, and a loosely fitted polycarbonate top (Fig. 1). Assembled units, shunt catheters, and polycarbonate molds were sterilized with ethylene oxide gas (130 min at 51–54°, 8 PSI). To promote alginate attachment to the chamber surfaces, chambers were treated with poly-l-lysine (PLL; 0.01 %) for 2 h at 37° and then washed in a bath of Milli-Q dIH_2_0 for 5 min. The proximal portion of a clinical ventricular catheter was mounted in the chamber. The PLL coating was confirmed using water contact angle measurements on a subset of chambers. To build the alginate scaffold, a polycarbonate mold (referred to as a “dam”) was positioned around a 32-hole (four rows of eight holes), barium impregnated, standard ventricular poly(dimethyl)siloxane (PDMS, silicone) shunt catheter (Medtronic, Goleta, CA, USA) using 4 % (w/v) low-melt agarose solution (Sigma) in sterile Milli-Q dIH_2_O to seal the spaces between the dam, catheter, and chamber (Fig. 2a, b).

#### Hydrogel components and chemistry

Alginate hydrogels/scaffolds were constructed by mixing a sterile stock solution of 6 % (w/v) calcium carbonate (Sigma), HBSS containing Ca^2+^, d-(+)-gluconic acid delta-lactone (GDL), and RGD prepared as a 5 % (w/v) stock. The final alginate concentration was 1.5 % (w/v) using a 0.54 Ca:COOH molar ratio following previously published methods [16]. To make the scaffold, the 6 % CaCO_3_ solution was vortexed to create a “slurry”, where the CaCO_3_ was evenly distributed through the solution. Thirty-four microliters of CaCO_3_ was pipetted into a 150 mg alginate aliquot and mixed, followed by the addition of 286 µL of Ca^2+^-HBSS. Working quickly, 30 µL of 214 mg/mL GDL was added to the alginate/calcium/HBSS mixture and stirred. The total mixture was then pipetted into the space between the dam the chamber and incubated in a cell culture incubator (37 °C and 5 % CO_2_) for 90 min to enable crosslinking (Fig. 2c, d). Following incubation and cell loading (Fig. 2d–e), the agarose and dam were carefully removed (Fig. 2f).

#### Loading scaffold with cells

Astrocytes were harvested from 80 % confluent cultures as described above, counted using a phase hemocytometer to determine the viable cell count, and re-suspended in 50 µL culture media. Cells were injected into the center of the gel using two 25-μL aliquots to yield 7.5 × 10^6^ cells/scaffold (total scaffold volume 500 μL, Fig. 2e). After a 5 min absorption period, complete culture media was added to the chamber to cover the scaffold, and the chamber was returned to the incubator for continuous culture. The culture medium was replaced three times per week.

### Design of 3D cortical cell model

Sterilized chambers containing the ventricular catheters were built as either “short” or “long” units (Fig. 1) allowing for different positions of the dam relative to the catheter holes (see chamber preparation). The short-chambered system (4-cm chamber length, 3.1-cm catheter length) was designed so that the alginate scaffold would cover half of the 32 catheter holes (Fig. 1a, c). The short-chamber system mimics situation when catheter movement or misplacement causes the shunt to be positioned with a proportion of holes residing within brain parenchyma. The long-chambered system (6-cm chamber length, 5.2-cm catheter length) was designed so all holes were at least 1 mm from the alginate gel (Fig. 1b, d). This mimics optimal shunt placement with all holes in the ventricular space.

A subset of experiments was performed with fluid flow to mimic physiologic flow of CSF in vivo [21]. These experiments used long-chambered systems and a three-channel peristaltic pump (Watson-Marlow 401U/DM3, Cornwall, UK), similarly to previously described methods [14]. In brief, media was pumped into the chamber, and upon chamber filling, media flowed from outside the catheter in through the catheter holes, similarly to CSF flow from the outside to the inside of the catheter in the ventricles (see Fig. 1d). Media only escaped the chamber by traveling through the catheter holes and inside the catheter. Briefly, flow rate (0.25 mL/min) and pulsation frequency (100 pulses/min) were set using pump specifications and 0.38-mm inner diameter double manifold peristaltic tubing (Watson-Marlow). The pump was positioned outside the incubator and attached to the assembled 3D culture system (chamber, pump, and medium reservoir) with barium-free medical grade silastic tubing (Advanced Medical Systems, UK). The tubing was sterilized with chambers using ethylene oxide. Prior to final assembly the tubing was primed with culture media.

### Gel characterization

#### Elastography

The viscoelasticity of alginate hydrogel, without cells, was determined using shear wave imaging, where the velocity of the wave through the gel is dependent on tissue elasticity [22]. A 200–400 Hz shear wave was launched with two consecutive ultrasound images through a 4 % agar 48 kPa gel with and without the inclusion of a 10 mm × 10 mm × 5 mm water-saturated, non-functionalized alginate hydrogel. The wave was imaged to determine the propagation rate/velocity of the shear wave through the gel. The median shear modulus of each sample was indirectly determined from calculations of the speed of the waveform through the alginate gels at each pixel. These calculations were performed in MATLAB using the relationship Ĝ = ρc(ω)^2^, where Ĝ is the shear modulus, ρ is density, c is the group speed of the shear wave, and ω is the angular frequency assuming gel incompressibility. The time of flight algorithm at each pixel was used to deduce a shear modulus map through the gel. Three median shear modulus measurements were computed for a small volume of interest in all alginate gels (n = 3). In this way, errors due to gel dimensions and boundary conditions were minimized.

#### Scanning electron microscopy

Pore structure and size was determined in the alginate gel using a JEOL Model JSM 7000F (JEOL USE, Peabody, MA, USA) scanning electron microscope (SEM) in secondary electron mode at 10 kV acceleration voltage. Prior to being gold sputter coated, samples were washed with distilled water to remove salts, frozen in distilled water, and lyophilized for 5–7 days.

### Fluorescent labeling and spinning disk confocal microscopy

Cell viability, cell attachment, and cell growth in the alginate, over the exterior of the catheter, and approximately 500 µm into the catheter holes, were measured at regular intervals from every 48 h to once weekly depending on experimental conditions and goals. A live/dead assay containing 2 µM calcein for live cells (emission/excitation peaks 494/517 nm) and 4 µM ethidium homodimer for dead cells (emission/excitation peaks 528/617 nm) (Invitrogen) was used to characterize cell growth and survival. Confirmation of cell distribution, numbers, and morphology was performed after fixation and labeling at either short (2-week) or long (8-week) exposures. Samples were fixed by adding 3–5 mL 4 % (w/v) paraformaldehyde to each chamber and incubated for 20 min. Using a dissecting microscope at 5×, catheters were cut longitudinally to measure astrocyte attachment in the catheter lumen and the holes oriented towards the bottom and top of the chamber. After rapid rinses with HEPES-buffered Hanks solution (HBHS), a 30 min incubation in 0.2 % Triton-X-100 (Sigma) in HBHS and a 30 min Image-iT FX signal-enhancer (Invitrogen) blocking step, astrocytes bound to catheters were labeled with 1:1000 monoclonal rat anti-glial fibrillary acidic protein (GFAP, Invitrogen) during incubations for 24 h at room temperature. After washing in HBHS, an aliquot of 1:200 polyclonal goat anti-rat Alexa Fluor 647 conjugated secondary antibody (Invitrogen) was applied and incubated for 24 h at room temperature. All catheters were additionally stained with 1:1000 CyQuant (Invitrogen) using 24 h incubations to identify cell nuclei. An Olympus IX81 inverted microscope with a DSU spinning disk confocal system, motorized xyz stage (American Scientific Instruments, Eugene, OR, USA), and Rolera EM-C2 camera (QImaging, Surrey, Canada) was used to acquire 800-µm × 800-µm × 500-µm confocal images using a 10× objective over 200 optical sections with a 2.4 µm step size. Datasets were collected in and around each catheter hole and on representative surfaces. To visualize the 3D datasets, maximum intensity projects were created (MetaMorph, Sunnyvale, CA, USA). Holes were referenced one through eight as they progressed from the catheter tip to alginate scaffold, with the hole closest to the catheter tip as hole one (Fig. 1). Data collection for holes one and eight was unobtainable due to silicone glue used to fix the catheter in the mounting chamber used for microscopy. This could not be avoided without risking migration of the catheter tip out of the microscope’s focal range.

### Cell quantification

The FARSIGHT toolkit (http://www.farsight-toolkit.org) was used to segment the fluorescent signal based on seed point detection [23–28]. In live-cell imaging, where the use of space filling dyes prevented us from separating overlapping cells, semi-quantitative results were achievable using volume rendering of the signal intensity.

Using images acquired with live/dead cell labeling, cells were observed to have several characteristic morphologies and organizations—round (small) cells likely in a state of movement or mitosis, cell clusters, large cells adherent to surfaces, cells lining the catheter hole walls, sheets of cells, and cells spanning across holes. A binary (yes/no) determination around each shunt catheter hole was used to identify an absence or presence of round cells, cell clusters, large cells, cells lining the catheter hole walls, cell sheets, and cells masses spanning across each hole (n = 24). Data around each hole were averaged to develop a semi-quantitative scoring system to analyze each shunt in each chamber (n = 4). Data are presented as the percent of experimental samples (shunts) observed with each morphologic characteristic, keeping in mind that the percentages are not mutually exclusive.

Post-hoc data was processed by quantifying the number of CyQuant-labelled cell nuclei using the FARSIGHT toolkit. Segmentation algorithms were used to segment all cell nuclei. These results were validated by visual inspection. Vaa3D Image Visualization and Analysis System was used as an additional quantitative tool to visualize samples in three-dimensional space [29].

### Statistical analyses

Statistical analyses were performed using SPSS (IBM Corporation; Armonk, NY, USA). The Anderson–Darling test for normality was used to divide data into parametric or non-parametric groups. Parametric data were analyzed using a paired two-tailed *t* test, a one-way analysis of variance (ANOVA), or a multivariate/repeat measures ANOVA with a Bonferroni correction. For all tests, a confidence interval was set at 0.95 (α = 0.05). Correlation between factors was determined by finding the Pearson correlation coefficient. In all repeat measures analyses, the Greenhouse-Geisser correction was used following a measure of sphericity. Following cases in which a paired t test or ANOVA was used, a post hoc Scheffe test was performed when the null hypothesis (no difference in the group means) was rejected. Non-parametric data were analyzed using the Kruskal–Wallis H test with an unplanned comparison of mean rank. To compare categorical data over time, data was preprocessed using MATLAB’s trapz function to find the approximate area under time-cell attachment curves. The resulting area under each curve was used for statistical analysis.

## Results

### Gel characterization

**Fig. 3:**
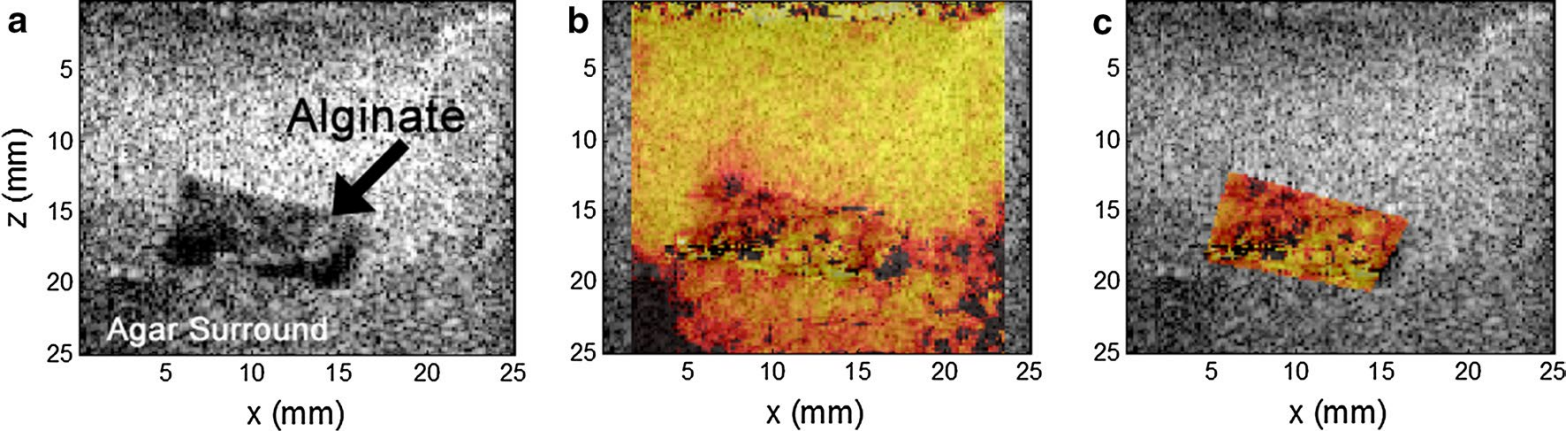
Scaffold mechanical integrity measured using ultrasound. Representative ultrasound images of an alginate scaffold imbedded in agar. **a** A raw ultrasound image illustrating an alginate scaffold embedded in agar. **b** A shear modulus map from a portion of the raw ultrasound image. **c** A shear modulus map of alginate scaffold after removing signal contributed by the agar embedding medium. These shear measurements were used to calculate the average median Young’s modulus. Measurements and calculations were made as described in the “Methods”

**Fig. 4:**
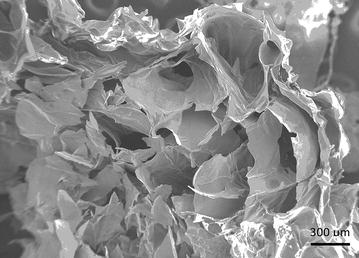
Alginate scaffold pore structure. Scanning electron microscopy image illustrating the pore structure of the alginate scaffolds. Sheets of alginate form cone-shaped pores. The narrowest diameters of these pores averaged 296.2 ± 83.8 µm (n = 5)

Two distinct observations were made to characterize alginate hydrogel scaffolds as the “brain” portion of the 3D cell culture system. First, the mechanical integrity of alginate scaffolds was measured to compare the modulus of our constructs with that reported for human brain parenchyma. The average median shear modulus was 4.54 ± 0.55 kPa (n = 5), where the standard deviation represents the spatial heterogeneity across all samples (Fig. 3). The standard deviation within each gel, whether built in the long or short-chambered system, averaged 0.09 kPa, suggesting that variability observed across gels was caused by inconsistencies in different scaffold constructs rather than in shear wave imaging. Assuming the shear modulus is approximately one-third the Young’s or elastic modulus (with near zero Poisson’s ratio), the tested alginate gels have an average calculated median Young’s modulus of 12.26 kPa. Secondly, SEM was used to assess the alginate hydrogel structure, particularly pore size and structure, to determine if these measurements were consistent with cell attachment and migration through the hydrogel to the catheter surfaces. Pores appeared conical with smooth surfaces. The narrower pore openings had an average diameter of 296.2 ± 83.8 µm (n = 5, Fig. 4). The smooth pore surfaces and relative large open pores are consistent with a structure that allows cell growth and movement.

### Astrocyte morphological characteristics and viability over time using the short-chambered design

**Fig. 5:**
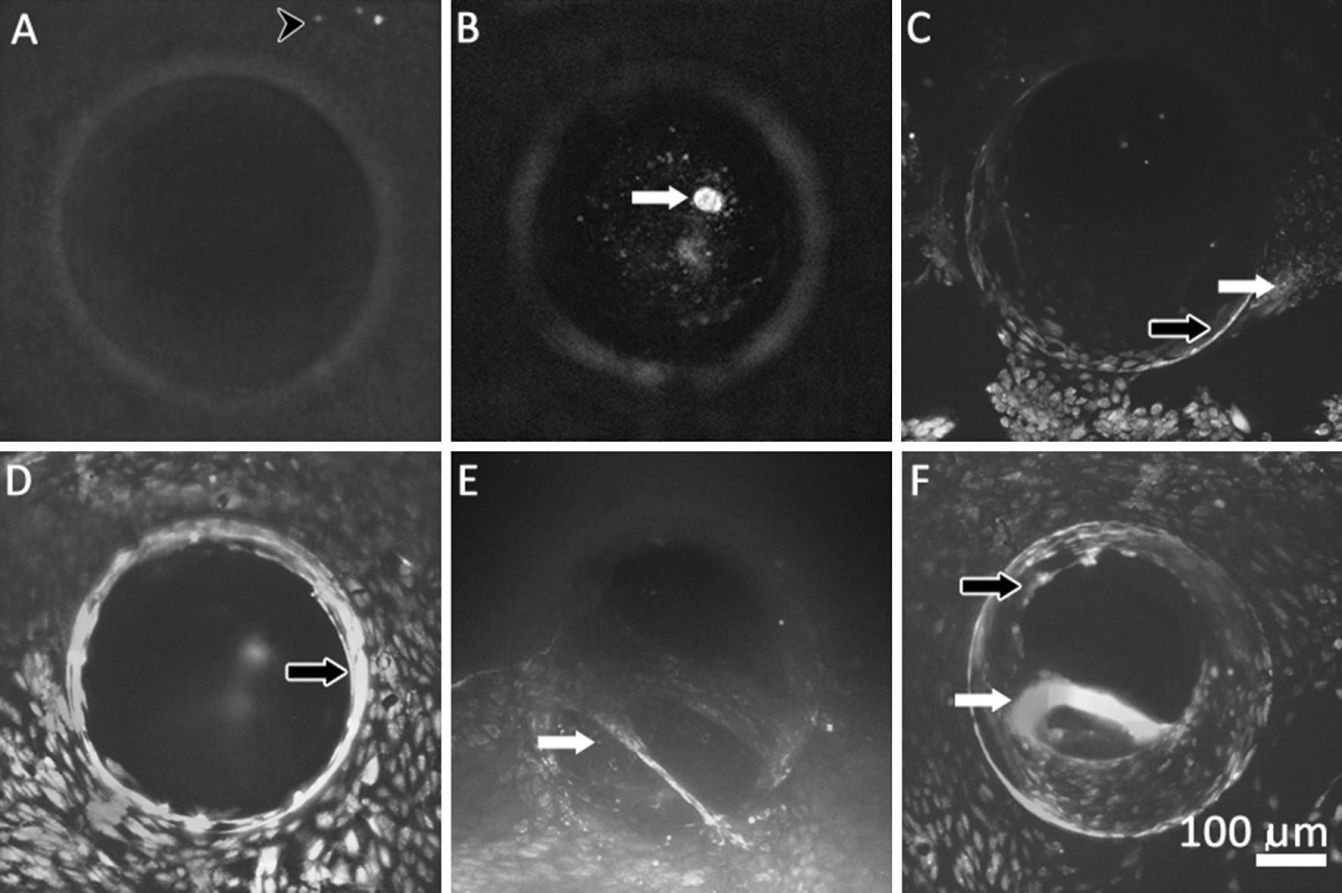
Representative 3D confocal imaging stacks displaying astrocyte morphological characteristics. Round, small cells likely in a state of movement or mitosis (**A**, *black arrowhead*) cluster together as if they were locally synchronized (**B**, *arrow*). Cells larger than the small, round cells of **A**, with more extended processes (**C**, *white arrow*) are likely adhered to the catheter surface. **C**–**F** Cells forming a network have been denoted as sheets of cells. Cells lining the walls of the shunt catheter holes (**C**, **D**, **F**, *black arrow*) seem to appear prior to cell masses crossing the hole to the opposite wall (**E**, **F**, *white arrow*). *Scale bar* represents 100 µm

Representative images of each classified morphological characteristic can be found in Fig. 5. It was clear that, over time, astrocytes emerged from the scaffold and attached to the outside of the catheter. Round cells were observed throughout 23 days of incubation and were observed in the alginate scaffold as well as on catheters where they were primarily observed near or in holes. We have defined ‘large cells’ (average diameter 19.17 ± 4.96 µm) as those in excess of 180 % the size of the typical astrocytes observed (average diameter 10.65 ± 2.76 µm). The number of large adherent cells generally increased over time. At longer culture times (>44 days of incubation) these hypertrophied astrocytes had a more cylindrical morphology and decreased in number. The large adherent cells were observed almost exclusively bound flat onto the shunt surface, whereas rounder/smaller astrocytes (average diameter 7.33 ± 2.09 µm) were observed in the shunt holes and in the alginate gel. Cells were also observed as clusters or sheets containing ten or more cells with interconnected processes. These were most frequently observed as clusters in the alginate scaffold or as sheets or clusters across or in catheter holes.

**Fig. 6:**
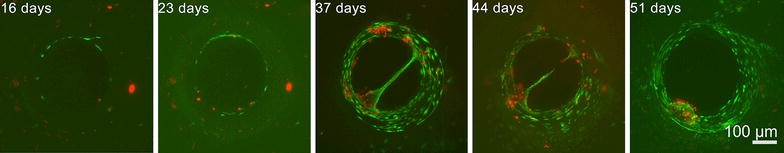
Astrocyte extension and retraction across shunt catheter holes. Representative images of a single catheter hole in the short-chambered system taken between 16 and 51 days of culture. The sample was stained to reveal viable (*green*) and non-viable (*red*) cells. Viable astrocytes are observed in holes as early as 16 days after the perfusion system was initiated. The dynamic growth (and retraction) of cells is illustrated in the 37-, 44-, and 51-day images when cells were observed bridging across the hole or lining the catheter hole wall. These images are maximum projections of 3-D confocal images. *Scale bar* represents 100 µm

**Fig. 7:**
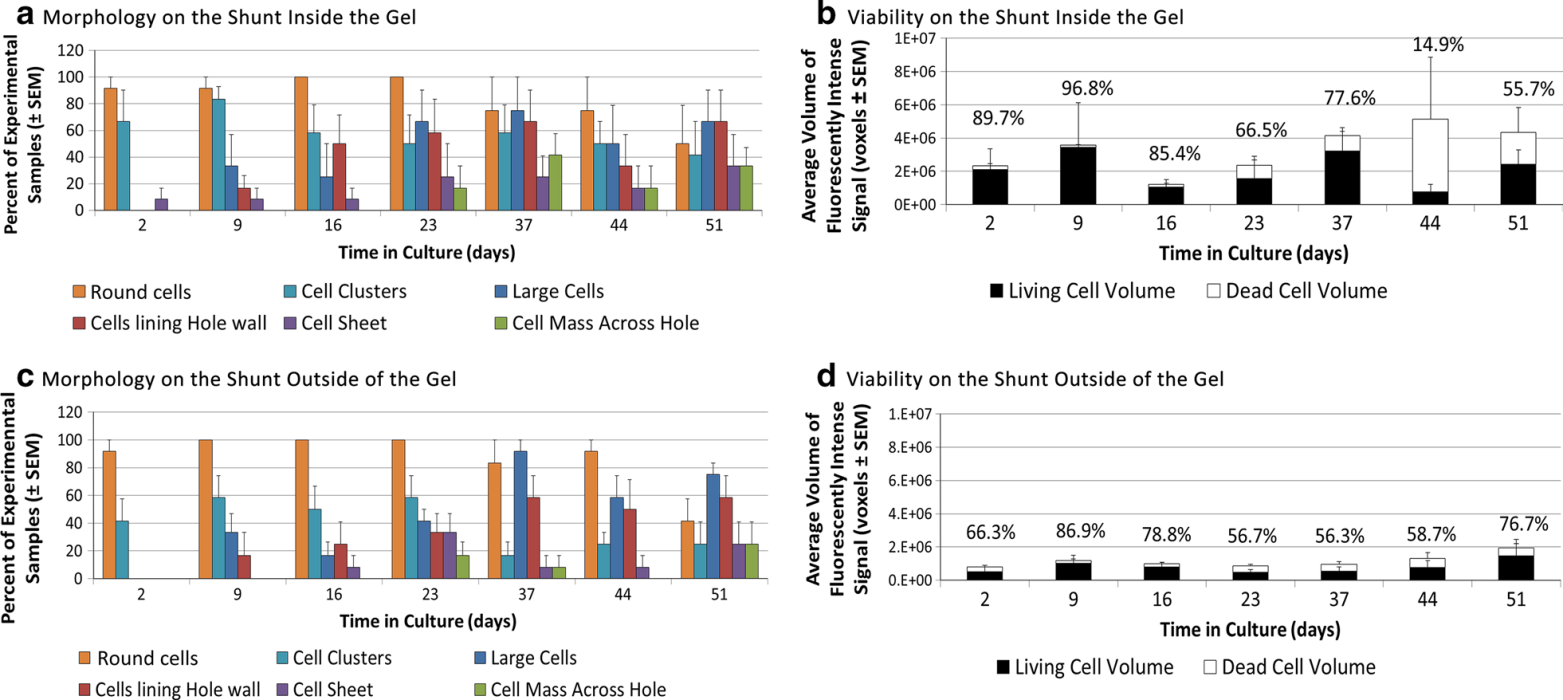
Astrocyte morphology and viability over time. Morphology (**a**, **c**) and viability (**b**, **d**) of astrocytes measured over time in a short-chambered system. *Each* *row* of figures presents data as observations made across the shunt catheter holes encased in the alginate gel (**a**, **b**), and holes outside of the gel (**c**, **d**). Morphology was scored by cell organization (single round cells, cell clusters, large cells, cell sheets and cells spanning shunt catheter holes). *Data* are presented as the total percent of shunts observed with each morphologic characteristic (n = 4), where the percentages are not mutually exclusive. Cell viability is scored by normalized fluorescent signal visualized using a live/dead cytotoxicity assay. The percent of viable cells out of the total cells is expressed above each time interval

Pearson correlation coefficient’s defined the association between morphological characteristics, independent of time, where positive correlations existed between large cells, cells lining the catheter hole wall, cells stretching across the hole, and cell sheets. Pairwise comparisons of astrocyte morphology over time in the short-chambered systems were performed using multivariate/repeat measures ANOVA (Table 1). Cell clusters and round cells were inversely related to these categories. The number of large cells increased with time in culture, reaching statistical significance (*P* < 0.05) from 2 days to all other time points and between days 16 and 37. A similar phenomenon was seen with cells lining the catheter holes as well as tissue spanning the catheter holes until day 44 when sudden decreases were observed (Figs. 6, 7). The number of cells lining the hole walls at each time point increased significantly (*P* < 0.05) compared to data at 2 days, and then thereafter from nine to 16 and to 23 days. Cells clustered more outside the gel on the shunt catheter than inside the gel at days 37, 44, and 51 (Fig. 7a, c). The average volume of fluorescently-intense live cell signal, indicative of the volume of living cells in the short-chambered system, was generally cyclical, where total cell volumes growth decreased at day 16 and 44 on the shunt hole inside or outside the scaffold (Fig. 7b, d). The degree of cell growth across holes was significantly greatest at day 37, but the ratio of living to dead cells fluctuated over time with the largest decrease in viable cells at 44 days when the presence of cells across holes trended downward.

**Table 1:** Statistically significant pairwise comparisons of astrocyte morphology over time (*P* < 0.05) using multivariate/repeat measures ANOVA with Bonferroni and Greenhouse-Geisser corrections

Time	Category	Category
2 days	Large cells	Cell clusters
		Round cells
	Cells lining hole wall	Cell clusters
		Round cells
	Tissue spanning across hole wall	Cell clusters
		Round cells
	Cell sheet	Cell clusters
		Round cells
	Cell clusters	Large cells
		Cells lining hole wall
		Tissue spanning across hole wall
		Cell sheet
		Round cells
	Round cells	Large cells
		Cells lining hole wall
		Tissue spanning across hole wall
		Cell sheet
		Cell clusters
9 days	Large cells	Tissue spanning across hole wall
		Cell sheet
		Cell clusters
		Round cells
	Cells lining hole wall	Tissue spanning across hole wall
		Cell clusters
		Round cells
	Tissue spanning across hole wall	Large cells
		Cell clusters
		Round cells
	Cell sheet	Cell clusters
		Round cells
	Cell clusters	Large cells
		Cells lining hole wall
		Tissue spanning across hole wall
		Cell sheet
		Round cells
	Round cells	Large cells
		Cells lining hole wall
		Tissue spanning across hole wall
		cell sheet
		Cell clusters
16 days	Large cells	Round cells
	Cells lining hole wall	Tissue spanning across hole wall
		Round cells
	Tissue spanning across hole wall	Cells lining hole wall
		Cell clusters
		Round cells
	Cell sheet	Cell clusters
		Round cells
	Cell clusters	Tissue spanning across hole wall
		Cell sheet
		Round Cells
	Round cells	Large cells
		Cells lining hole wall
		Tissue spanning across hole wall
		Cell sheet
		Cell clusters
23 days	Large cells	Round cells
	Cells lining hole wall	Round cells
	Tissue spanning across hole wall	Round cells
	Cell sheet	Round cells
	Cell clusters	Round cells
	Round cells	Large cells
		Cells lining hole wall
		Tissue spanning across hole wall
		Cell sheet
		Cell clusters

Based on estimated marginal means

**Fig. 8:**
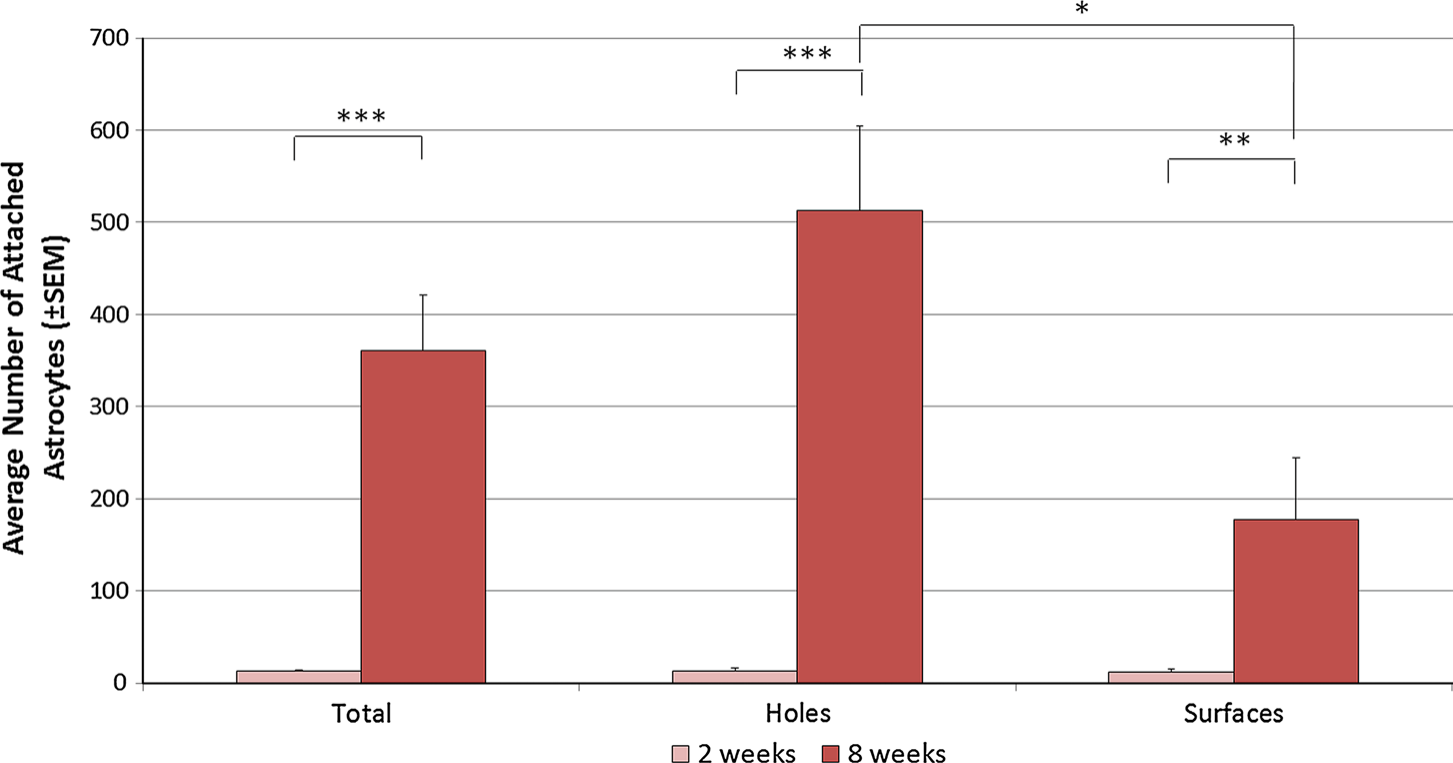
Acute and chronic astrocyte attachment on/around shunt catheter holes and surfaces. Number of astrocytes attached to the shunt catheter surface in the short-chambered system using post hoc cell nuclei counting at 2- and 8-week incubations. (*P < 0.05, **P < 0.01, ***P < 0.001). Surface area was not accounted for in these estimates (see “Discussion”)

To confirm our findings that cell attachment generally increased over time, we quantified the number of cells attached to the shunt catheter at varying fixed end points (14 and 56 days). Similarly to analysis of viable cells over time, a significant (*P* < 0.001) difference in total cell attachment and growth was observed between experimental endpoints 14 and 56 days (2 and 8 weeks, Fig. 8).

### End point analysis of cell attachment on varying shunt surfaces

Using the short-chambered system at fixed endpoints (14 and 56 days in culture), we compared astrocyte attachment and growth at varying locations within the system (i.e. on the shunt catheter in and out of the gel, on the catheter’s top and bottom halves, and in shunt catheter holes and on shunt catheter surfaces between holes). There was a significant increase in cell attachment in and around the catheter holes and on shunt surfaces (between holes) from 14 to 56 days (*P* < 0.001 and <0.01, respectively). By 56 days (8 weeks), significantly more cells were attached to holes than to catheter surfaces (*P* < 0.05). There were no significant differences between the numbers of cells on the top and bottom portions of catheters (gravity-dependent variation). There were also no significant differences in cell attachment inside versus outside of the gel, but cell numbers trended upward closest to the gel.

### Effect of shunt position: variance between short- and longchambered design

Total cell attachment on shunt catheters was dependent on the initial positioning of the shunt in reference to the gel. That is, data collected using the short-chambered design, where half of the shunt’s ventricular catheter holes were covered by the gel, had significantly less total shunt-cell binding per square millimeter than data collected using the long-chamber design, where none of the shunt’s ventricular catheter holes were initially covered by the gel (*P* < 0.001). The key variance in total cell attachment appeared at hole 5, where there was significantly more cell attachment on shunts in the long-chambered design than on shunts in the short-chambered design (*P* < 0.05, data not shown). Differences in attachment at all other holes were insignificant.

### Flow dependent attachment and growth using the long-chambered design

**Fig. 9:**
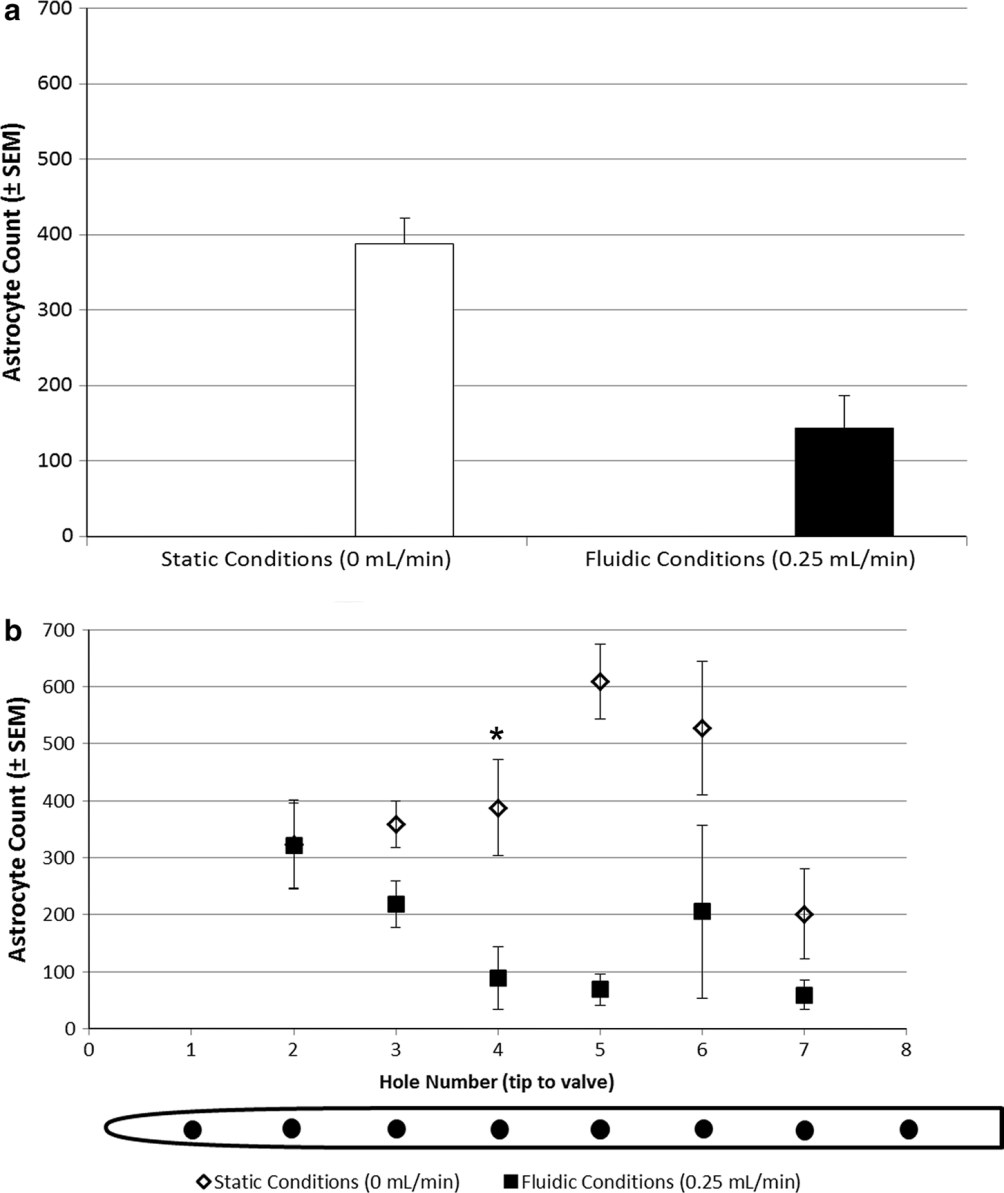
Introduction of physiologic fluid flow. Total (**a**) and per hole (**b**) cell attachment under static conditions and under physiological flow after 18 h in the long-chambered system. A trend in the data show a reduction in total cell attachment under conditions of flow and at each hole (*P < 0.05)

A subset of long-chambered units (n = 3) were used to observe the effects of flow-induced shear at physiologic flow rates on cell attachment and growth. After exposure to peristaltic flow for 18 h, there was a qualitative decrease in the total number of attached cells, but this difference was not significant compared to the degree of attachment before flow (*P* = 0.086, Fig. 9a). When exposure to flow was analyzed with respect to hole location, there was a significant difference due to the effect of flow and added exposure time (18 h) at hole four (*P* < 0.01, Fig. 9b).

## Discussion

The complex mechanisms of shunt obstruction are multi-faceted and poorly understood requiring systematic study with single variable manipulation, in order to appreciate how and why shunt failure occurs. Data presented here demonstrate that astrocytes may preferentially attach to the walls of the catheter holes resulting in accumulations of additional cells. Cell accumulation can result in cell sheets or cell masses completely occluding the catheter holes (Figs. 5, 6). We propose that shunts fail after becoming obstructed with attaching glial cells, creating a substrate for more glia or other cells and tissues (e.g. choroid plexus) to secondarily bind to the shunt and block CSF flow. Our in vitro system models this inflammatory response by replicating the interface between the parenchyma and shunt using an astrocyte-seeded 3D alginate scaffold surrounding a portion of the shunt’s ventricular catheter.

### Physical properties of the 3D alginate matrix modeling the parenchyma

Alginate, a natural polysaccharide used extensively as a hydrogel synthetic extracellular matrix (ECM), has numerous free functional groups to enable inclusion of biochemical signaling molecules, like the tripeptide RGD [15]. Unlike other scaffold materials, alginate does not require the addition of cytotoxic agents to crosslink. In this study, an alginate matrix was seeded with astrocytes at a high density to mimic the astrocyte density in human cerebral cortex. The hydrogel held cells in 3D space while providing adequate space for cell attachment, proliferation, cell movement, and normal cell metabolic function. Using our protocol, the alginate gel had a similar pore macrostructure to 5.0 % (w/v) gelatin foam made to mimic ECM, but lacked the collagen-like nanofibrous architecture which may contribute to cell attachment and growth [30]. The relatively large pore size of the alginate matrix was sufficient to allow astrocytes to extend their processes and to maintain cell clusters within an individual pore (Fig. 4). Although the morphology of the cells pointed to near-normal cell function, the rate of cell migration onto the shunt surface was not directly comparable to in vivo preparations. Future work should include a thermally-induced phase separation technique and/or porogen leaching to create a nanofibrous structure with interconnected macroporous architecture to more accurately mimic cortical tissue. The addition of proteoglycans may also more accurately mimic in vivo conditions [31, 32].

Tissue/gel elasticity plays an important role in ECM assembly, cell spreading, and cell motility; therefore, our model should include a gel with similar elasticity to tissue. Anchorage-dependent cells, like astrocytes, are particularly responsive to the mechanical properties of their matrix, and preferentially attach to stiffer surfaces [33]. The stiffness of brain tissue varies in the literature, probably because of a dependence on acquisition method (e.g. acquisition frequency), mathematical assumptions, the specific brain region assayed, and the presence of any pathology. Young’s moduli of whole brain tissue can range from of 0.5–37 kPa, acquired from mathematical modeling [34], magnetic resonance elastography (MRE) [35, 36], and shear wave imaging [22]. Reported shear moduli also varies, ranging anywhere from 2.5 to 15 kPa for white matter and 2.8–13 kPa for gray [37]. Comparing data acquired from shear wave imaging only, we see that our gel (measured shear modulus 4.54 ± 0.55 kPa, calculated Young’s modulus 12.26 kPa) falls within this large range, but is less stiff than Mace et al’s shear wave measured shear moduli of whole brain (12.3 ± 0.7 kPa) and of cortical tissue (12.9 ± 1.3 kPa) [22].

We chose to maintain these mechanical properties for two reasons. First, the measured shear modulus does fall within the wide range of reported shear moduli. Second, recent evidence indicates that brain parenchyma modulus in hydrocephalic patients may be considerably lower than originally predicted, and lower than non-pathologic brain parenchyma due to pathological factors that influence brain compliance including tissue compression against the rigid skull, and the chronic white matter damage associated with hydrocephalus [34]. The high stiffness of the shunt catheter relative to brain parenchyma may contribute to cell migration and/or proliferation on the catheter surface. Over time, the cell population on the catheter surface increases, as does the number of cells in the catheter hole wall where surface roughness is higher [38]. In preliminary results in our lab using the same alginate preparation but with cells mixed into the gel rather than injected, the shear modulus (11.68 ± 2.78 kPa) is similar to the shear modulus of whole brain and cortical tissue. Future work will closely examine this relationship in an effort to improve our relevance to in vivo hydrocephalic conditions.

Finally, it is important to note that Ca^2+^ cross-linked alginate hydrogels lose mechanical properties over time in vitro, presumably due to slow loss of crosslinking Ca^2+^ ions into the culture medium [16, 39]. This loss of scaffold integrity may have contributed to the observed increase in cell death after 23 days post-seeding (see cell quantification discussion below).

### Cellular properties of the 3D alginate matrix

The morphology and organization of cells was as expected, where some astrocytes were found adherent to the shunt catheter surface (‘large’ cells), and others (‘round’ cells) were imaged as they were likely navigating toward a point of attachment on the gel or catheter (Fig. 7). Differences in morphological characteristics were more pronounced in the first 16 days of experimentation, indicative of astrocyte activity and/or a sustained evoked inflammatory response to the alginate gel or the shunt catheter. Interestingly, the number of adherent ‘large’ cells appears to wax and wane over time in a cyclical progression toward shunt catheter obstruction. The gradual increase, sharp decrease, and subsequent increase in viable cells observed from day 37 to 51 in our chronic experiments, while initially counterintuitive, may yield new insight into shunt obstruction at the sight of the ventricular catheter. Initially (48 h to 37 days post seeding), the average volume of space occupied by viable cells, the presence of cells lining the hole walls, the presence of a sheet of cells, and the presence of cell growth across holes generally increases regardless of catheter hole location (in or out of the gel), probably due to cell proliferation, cell spreading, and cell migration. After 44 days in culture, the volume of viable cells decreases, the volume of non-viable cells dramatically increases, the presence of cells lining the catheter holes decreases, and the presence of tissue across the holes decreases. That is, the viable cell masses appear to retract instead of spread, possibly because of chemical cues (contact inhibition, internal tension, a lack of available ECM, a change in cell shape) or physical cues (distance between adhesive hole walls). Then, after 51 days in culture, this retraction is overcome and viable cell attachment returns. This is coupled with a reduction in round cells and an increase in cells lining the hole walls, cell sheets, and tissue across holes. This cyclic pattern of progression was seen repetitively across samples, but we must acknowledge that some experimental caveats, like the static environment, number of media changes, and lack of other cell types could contribute to these findings. If this finding is physiological, however, this phenomenon may begin to depict how shunt catheter obstruction by astrocytes occurs. We can speculate that perhaps the rate of this cyclical cell viability is different across patients, explaining, in part, why shunt obstruction occurs at different rates in patients despite similar cell types found obstructing shunt catheters. Impeding the rate of cell spreading by disrupting the receptor-ligand interactions or by enhancing retraction through chemical or physical means may be potential methods to reduce the rate of shunt obstruction. Work comparing this model to catheters explanted from patients is ongoing in our lab and will help validate potential mechanisms underlying cell-catheter interactions.

Post-hoc analysis of the luminal surface of the catheters confirmed the live/dead assay data, with cell attachment and growth generally increasing over time, with significantly more astrocytes bound to shunts incubated for 8 weeks compared to 2 weeks, regardless of using the short- or long-chambered systems (Fig. 8). After 8 weeks, astrocytes bound preferentially to shunt catheter holes as compared to shunt catheter surfaces, perhaps because of greater surface roughness of the hole walls [40–43]. However, our cell attachment data were collected by analyzing confocal stacks of the same x,y space capturing all attached cells in z-space. Through the holes, the surface area for cell binding was at least two times greater than the surface area of the same x,y space on the shunt surface because of the hole’s z height. Accounting for this difference minimizes any preferential binding on the shunt holes to near zero. If we assume that attachment on the shunt hole walls leads to an extension of tissue across the hole, flow would impeded throughout the hole, independent of where the cell mass was in z-space. So, this surface area normalization may not be clinically relevant.

Interestingly, there appeared to be more cell attachment in our long-chambered design than our short-chambered design where holes were in the gel modeling the brain parenchyma. This is counter-intuitive, since suboptimal shunt placement or shunt movement has been shown to increase failure rates. This may be because the long-chambered design contained a larger volume of nutrient-rich cell culture media than the short-chambered design. Fortunately, introducing flow into the model eliminated any issues of cell starvation and improved the physiological relevance of the system.

### Flow-dependent cell attachment

There are very few models that try to interpret the effects of flow on cell attachment to improve hydrocephalus treatment. Astrocytes are an appropriate cell type to analyze flow-dependent changes in attachment because flow has been shown to dramatically influence the degree of attachment, and to a lesser extent astrocyte morphology [14]. In our previous model, the hydrocephalus shunt catheter bioreactor (HSCB), we showed that suspended astrocytes have an increased binding propensity with increasing flow-induced shear stress (shear stress from 0.0039 to 0.0134 Newton per square meter, N/m^2^), presumably because of (1) a rate-dependent change in the cell receptor to bound protein ligand contact time and contact incidence; and/or (2) a biochemical change in astrocyte properties [14]. This finding was threshold dependent, however, and a relatively large shear stress (0.2409 N/m^2^) generated enough force to overcome any cell-protein interactions generated from flow [44]. In the present study, increasing shear stress at low levels by introducing physiological flow (0.0089 N/m^2^ through the catheter lumen, 0.0075 N/m^2^ through each catheter hole assuming that flow is dispersed equally through each hole) appears to decrease attachment after approximately the same time period (18 h)—perhaps because an increase in flow disrupts contact between the attached cells and the adsorbed protein ligands (Fig. 9). Parsing the data into cell attachment around each hole, we can see that hole five appears to have the most cell attachment in both the static and fluidic long-chambered systems. Because results are similar to the static cultures and because we know that flow is not evenly distributed across the holes [45], we can predict that attachment is not directly associated with calculated flow-induced shear in this model.

The HSCB model and the flow model in the present study, model quite different mechanisms of ventricular catheter obstruction: the former measures how flow modulates the act of single-cell attachment, whereas the latter measures how physiologic flow disrupts cells already attached to the catheter and migrating down the catheter shank. Both of these mechanisms are quite relevant to shunt obstruction; we must prevent both. Future work must include analysis of the chronic effects of flow, and should also examine how flow modulates the act of whole-tissue (e.g. choroid plexus) attachment without static incubation preceding flow. It is necessary to delineate the difference between cell attachment and growth because flow, surface chemistry, and the architecture of the catheter may influence these factors independently, as has been shown [10, 38, 46, 47]. Future studies should include detachment experiments to record the initial strength of cell adhesion, cellular morphological assessment, and quantification of proliferation over time [48]. This model can be modified to incorporate other cells in the 3D scaffold, like microglia, blood-borne macrophages, and necrotic cellular debris and by combining with a previous model using cells suspended in a CSF-like solution, like astrocytes [14], microglia, bacteria, red blood cells or platelets, tumor cells, or necrotic cellular debris. Ongoing studies in our lab are experimenting with the incorporation of rat choroid plexus explants and ependyma. Finally, we can examine the role of these cells, cell masses, and tissues on shunt valve obstruction by using clinical hardware in vitro.

It has been shown that irritation and inflammation of periventricular tissue is a source for reactive cells to infiltrate the catheter [10, 11, 13, 49, 50]. The irritation of the parenchyma and the resulting astrocytic response is modeled here. Our model can also incorporate important pathophysiologic changes such as fluctuations in intracranial pressure or protein concentration both in the gel (modeling the parenchyma) and in the solution (modeling CSF). Other cell types can also be introduced by themselves or as a co-culture with astrocytes. Choroid plexus infiltration causing shunt failure has not been addressed in this model, but serves as an area of future work for our lab. The development of cerebral organoids, which could potentially recapitulate choroid plexus may be an avenue worth exploring in this endeavor [51]. An extensive analysis of resected catheters from patients provides supporting observations that the growth of cells in our 3D culture system closely mimics what we observe on resected catheters and supports our proposal that astrocytes and microglia migrating from the brain parenchyma may be predominately responsible for cell obstruction of catheter holes.

## Conclusions

Alone or in combination with previously-published cell culture models of shunt obstruction, this model serves as a relevant test bed to analyze mechanisms of shunt failure and test catheter modifications that will prevent cell attachment and growth. Of particular relevance, we found that: (1) the stiffness of the three-dimensional cell-loaded “parenchyma” falls in a similar range to brain; (2) astrocytes are viable and migrate out of the gel similarly to how cells are hypothesized to migrate from brain parenchyma; (3) astrocyte attachment around shunt catheter holes appears to extend and retract across the hole in a cyclical pattern of attachment leading to obstruction; (4) the three-dimensional scaffold system can be incorporated into a fluidic cell culture system to mimic some physiologic parameters seen in the brain’s ventricular space.

## Abbreviations

CSF: cerebrospinal fluid
w/v: weight-to-volume
MES: 2-(*N*-morpholino)ethanesulfonic acid hydrate
RGD: arginine-glycine-aspartic acid
PLL: poly-l-lysine
PDMS: poly(dimethyl)siloxane
GDL: d-(+)-gluconic acid delta-lactone
HBHS: HEPES-buffered Hanks solution
GFAP: monoclonal rat anti-glial fibrillary acidic protein
HSCB: hydrocephalus shunt catheter bioreactor
